# Exploring tutor perceptions and current practices in facilitating diagnostic reasoning in preclinical medical students: Implications for tutor professional development needs

**DOI:** 10.15694/mep.2018.0000106.1

**Published:** 2018-05-22

**Authors:** Jane Elizabeth Keir, Shannon Lea Saad, Lorna Davin

**Affiliations:** 1 The University of Notre Dame Australia; 2 The University of Notre Dame Australia; 3 The University of Notre Dame Australia

**Keywords:** Clinical Reasoning, Tutor development, Educational Strategies, Preclinical

## Abstract

This article was migrated. The article was marked as recommended.

**Introduction:** This study explores tutors’ experience of teaching diagnostic reasoning (DR) - a key component of clinical reasoning - to build understanding into the use of explicit strategies in facilitating development of DR skills in preclinical medical students.

**Methods:** A qualitative, interpretive study was undertaken with 14 preclinical problem-based learning tutors who participated in semi-structured interviews. A thematic analysis was conducted to identify key factors that influence students’ learning of diagnostic reasoning.

**Results:** Tutor dispositions towards facilitating learning of DR were variable in this study. Explicit strategies to teach DR were thought to exert positive influences on the development of DR skills, through improving student knowledge and reducing potential error. The advantages of using explicit strategies to teach DR outweighed the perceived difficulties identified in this context. Explicit strategies may need modification for preclinical students and the focus should be on building knowledge of classic presentations and developing metacognitive awareness.

**Conclusion:** The use of explicit educational strategies will contribute to facilitating preclinical student learning of DR skills. Tutor professional development is a key component in the successful implementation of these strategies.

## Introduction

Clinical reasoning (CR) is an essential skill for health practitioners. Poor CR skills are responsible for a significant percentage of medical error ( Croskerry, 2009), particularly diagnostic error ( Nendaz and Perrier, 2012). Clinical reasoning (CR) encompasses a variety of cognitive processes involved in a clinical encounter, including data gathering and analysis, hypothesis generation, retrieval of stored knowledge, and diagnostic and management decision-making. Diagnostic reasoning (DR) refers to the component of CR relating to making a diagnosis. This study has focused specifically on DR (rather than CR generally), as this is an early step in the reasoning process particularly relevant in the preclinical setting. Whilst some of the literature cited refers to CR generally, it has been used because of its relevance to the DR component of CR.

The CR process is often poorly understood by clinicians ( Frick & Bezuidenhout, 2014). Because of the tacit and complex nature of CR, it is often invisible to students and therefore difficult to learn if implicit teaching methods are used ( Delaney & Golding, 2014). Traditionally, the development of DR skills was thought to take place during clinical placements. The literature, which predominantly focuses on students in clinical contexts, suggests CR should be taught *explicitly* ( Eva, 2004; Eva et al, 2007; Ryan & Higgs, 2008; Atkinson et al, 2011). Many authors also suggest teaching of CR should start *early* ( Kassirer, 2010; Schmidt & Mamede, 2015; Modi et al, 2015). CR and DR have increasingly been introduced into the preclinical curricula of medical schools (e.g. with problem-based learning and, more recently, specific CR courses), but there is limited research regarding the efficacy or associated challenges of introducing DR in the preclinical setting.

### Theoretical perspectives

Expert CR can be thought of as a mix of critical thinking, reflection and creativity, influenced by knowledge, intuition and experience ( Frick & Bezuidenhout, 2014). Because of the complexity of the CR process, most clinicians use multiple, combined reasoning strategies ( Eva, 2004; Norman, 2006). Various theoretical perspectives have been studied over the last 40 years, in an attempt to understand CR. Initially the focus was on problem-solving and general strategies with the development of dual process theory, but more recently, the central role of knowledge and how it is stored has emerged as an influential factor ( Norman, 2005).

Dual process theory as it applies in medicine is the concept of non-analytic and analytic reasoning, and it is widely accepted that that these two approaches play a key role in DR ( Eva, 2004; Kassirer, 2010; Bowen, 2006; Norman, 2005). Non-analytic reasoning is fast, intuitive and relatively effortless, compared with analytic reasoning which is slow, systematic and requires high cognitive effort. Experts are thought to use non-analytic reasoning (e.g. pattern recognition, where a set of clinical findings is easily matched with a previously seen case) predominantly, but resort to an analytic approach (e.g. hypothetico-deductive reasoning involving hypothesis testing) if presented with unfamiliar, ambiguous or particularly complex scenarios. Novices, in contrast, have less clinical experience on which to draw and spend more time using analytic reasoning ( Croskerry, 2009).

The way in which knowledge is organised and stored in long-term memory, and therefore how easily it can be accessed, is thought to be crucial in the development of expertise in CR ( Cutrer et al, 2013). Script theory ( Charlin et al, 2007), which asserts that knowledge is structured and stored in a way to make it accessible, provides a cognitive basis for understanding this process. Initially, students develop extensive causal mechanisms to link pathophysiology and clinical consequences. With experience, there is a restructuring of this knowledge and ‘encapsulation’ of biomedical and clinical knowledge into higher-order, summarised concepts. Further reorganisation and the addition of enabling conditions leads to the development of ‘illness scripts’ ( Schmidt et al, 1990, p. 614). Illness scripts include prototypes and exemplars or instance scripts (real cases stored in memory that can be drawn on for pattern recognition) and allow stored knowledge to be more readily retrieved ( Schmidt and Rikers, 2007). Earlier forms of stored knowledge (e.g. causal mechanisms) are still available to be used if necessary ( Schmidt et al, 1990). Scripts are activated and used to guide data gathering in DR, with the aim of identifying a script that most closely fits the clinical findings. The most likely activated script becomes the provisional diagnosis ( Charlin et al, 2007).

A third theory, cognitive load theory, assumes the brain has limited working memory and supports the necessity to store knowledge in easily retrievable packages in long-term memory ( Yu et al, 2014). This adds further weight to the importance of script theory and how knowledge is stored.

The relevance of these theories in practice are now discussed.

### Clinical and educational implications

During a patient encounter, a clinician may recognise specific features of the case as matching previously-seen cases. If not, they make a mental representation of the case by using a variety of techniques, including use of ‘abstract semantic qualifiers’ - terms that often have a paired opposite e.g. acute/chronic, intermittent/continuous ( Bowen, 2006, p. 2221) - and comparison with prototypes or illness scripts ( Schmidt and Rikers, 2007). These methods assist with generation of hypotheses, which guide further data gathering, and determine which hypotheses match the case or need to be discarded. Most diagnostic error is thought to be related to problems with DR, particularly faulty data gathering, analysis and verification of hypotheses ( Cutrer et al, 2013).

A clinician’s thinking can be affected by a variety of biases and shortcuts or heuristics ( Croskerry, 2003). Some of the more common cognitive biases are listed in Table 1.

**Table 1.** Common Cognitive Biases

Name of Bias	Description
Availability	Bias due to the influence of recently seen clinical cases
Representativeness	Over-dependence on probabilities
Confirmation bias	Information is only sought to confirm a pre-existing belief
Premature closure	Quick decision-making before all information has been considered

Clinician factors such as positive or negative feelings toward the patient, fatigue and overconfidence have also been recognised to influence reasoning and contribute to cognitive bias (Pinnock and Welch, 2014). Biases and shortcuts increase in conditions of uncertainty or time pressure. Some heuristics are helpful (e.g. ‘worst-case scenario’, with consideration of potentially life-threatening or serious, not-to-be-missed diagnoses), but others can lead to premature closure or diagnostic error ( Croskerry, 2003).

Several explicit teaching strategies have been found to improve diagnostic reasoning skills in the *clinical* setting:

• ‘Making thinking visible’ (as described by Delaney & Golding, 2014, p. 2) involves the clinician identifying their own thinking steps, repackaging them into short, repeatable actions or thinking routines, articulating them to students and encouraging students to use them frequently and regularly.
• ‘Problem representation’ involves using ‘abstract semantic qualifiers’ to make a 1-2 sentence summary of the key features of a case ( Bowen. 2006, p. 2221; Cutrer et al, 2013, p.253; Norman, 2005). This improves data processing, facilitates access to stored knowledge of cases or diseases (including pattern recognition and illness scripts) and subsequently helps students generate more likely hypotheses.
• Using classic presentations, or prototypes, and considering which findings in the case match the typical presentation, can serve multiple purposes. The prototype can be used to guide further data acquisition and enable prioritising of hypotheses. It can also help students develop rudimentary illness scripts, which can then be further enhanced as they encounter less typical presentations ( Bowen, 2006).
• Encapsulation can be facilitated by using concept maps to link clinical and basic science concepts ( Huang et al, 2014).
• Using prior knowledge to justify hypotheses and comparing and contrasting diagnoses will assist the development of illness scripts ( Modi et al, 2015; Bowen, 2006; Schmidt & Mamede, 2015). This process should involve the identification of defining and discriminating features.
• Metacognition, or thinking about one’s thinking ( Croskerry, 2003), plays a large part in DR ability. Increased metacognitive awareness should be encouraged to help learners slow down, reassess and reduce error due to bias or heuristics ( Rencic, 2011; Modi et al, 2015; Atkinson et al, 2011). Understanding that cognitive biases and errors can influence reasoning (Pinnock & Welch, 2014) and employing specific strategies to minimise these influences should lead to better decision making and less error ( Kassirer, 2010).

The various theories of reasoning described previously suggest many ways we can help students improve their knowledge and facilitate development of their DR skills. This research aims to explore the influence of explicit strategies to teach DR on the development of DR skills in preclinical medical students, from a tutor’s perspective. Exploring the experience of tutors (including their current practices and perceptions) will inform our understanding of the impact of explicit DR strategies and thereby enhance teaching and learning practices in this field.

Two research questions informed this study:

• How do tutors perceive that explicit teaching of DR reasoning strategies influences diagnostic reasoning skills in preclinical medical students?
• What do tutors perceive are facilitating factors and barriers to teaching and learning of DR skills in preclinical medical students?

## Methods

### Participating University

The University of Notre Dame School of Medicine, Sydney, Australia offers a four-year, graduate entry medical degree, where problem-based learning (PBL) is supported by lectures, tutorials and workshops. An ethics proposal was submitted, and approval obtained from the Human Research Ethics Committee of The University of Notre Dame - Reference number 016098F.

### Study Participants

The 14 medical doctors and teaching clinicians who participated in this study were tutors to second year medical students and were recruited by email sent to the entire cohort (16) of tutors of second year students. Electing to use participants from one university allowed exploration of the research questions purely from a clinician perspective, as other medical programs often use PBL facilitators with a non-medical background, who could have a significantly different perspective. The research was confined to tutors of second year medical students as this is the year immediately prior to entry into the clinical setting.

### Design and procedure

A qualitative, interpretive study, grounded in a constructivist paradigm, was undertaken. The aim was to build knowledge by exploring and seeking to understand the *individual* experiences and meaning making of the tutors ( Philpott & Batty, 2009).

Participants were asked to complete an initial Likert-style survey regarding their knowledge of, skills in and attitudes towards teaching diagnostic reasoning, and identify whether they would be willing to participate in a further interview if invited. This screening survey allowed us to understand the background characteristics of the group and ensure the participants interviewed had a range of experience and attitudes. Selected participants then underwent a semi-structured, one-on-one taped interview of 30-40 minutes’ duration. A decision was ultimately made to interview all 14 participants to develop as rich an understanding of the issues as possible, however saturation of data was achieved after the eleventh interview. All interviews included discussion of the following topics:

• the tutor’s understanding of DR processes
• the tutor’s approach to teaching DR
• the tutor’s perception of the usefulness of their current DR teaching strategies
• barriers identified by the tutors to teaching and learning of DR in the preclinical setting
• comparison with, and opinions on, the following 5 evidence-based strategies that have been shown to be useful in professional development in facilitating DR in the clinical setting:

• ‘making thinking visible’
• ‘problem representation’
• use of prototypes to support hypotheses
• comparing and contrasting hypotheses using defining and discriminating features
• metacognitive techniques.

The interviews were transcribed, coded and a thematic analysis performed, identifying patterns or themes in participants’ responses and analysing these in relation to the theory underpinning this study. Consideration was given to the entire data set. A deductive or ‘top-down’ approach ‘driven by the researcher’s theoretical and analytic interest in the area’ ( Braun & Clark, 2006, p. 84) was used. Themes were identified using a latent approach (Boyatzis, as described by Braun & Clark, 2006) looking beyond the semantic content of the data and considering underlying assumptions and ideas.

A member check was performed in order to improve the credibility of the research, with three participants asked to advise if they thought the results were credible. This also increased the agency of the participants ( Varpio et al, 2017).

## Key Findings

### Demographic overview

The mean teaching experience of participants was 9.85 years with a median of 8.5 years (range 1 to 25 years). Eight of the 14 tutors had formal teaching qualifications but, of those, only two had a qualification that included a specific clinical reasoning component. Thirteen had completed a traditional lecture-based medical degree and one was from a problem-based learning background. There were five male and nine female participants.

### Thematic analysis

The thematic analysis of the tutors’ perceptions illuminated four over-riding themes ( Table 2), which are discussed in more detail below:

**Table 2.** Summary of Key Themes

1. Tutor dispositions towards facilitating learning in DR 2. Tutor perceptions of established evidence-based strategies to teach DR 3. Relationships between subject knowledge and DR 4. Student readiness to engage in DR.

1. Tutor dispositions towards facilitating learning in DR

The research highlighted the vital role the tutor plays in facilitating development of DR skills.

Tutor awareness had a significant influence on facilitation style. Despite being experienced clinicians and educators, only half of the participating tutors could articulate their own DR processes, and significantly, they did so using general concepts rather than articulating specific steps in the process:

*I will use pattern recognition, and if for some reason it doesn’t advance anywhere, then I’ve got to come back and be more thorough, going through a more set process of history or examination* (Tutor G)

All tutors were teaching analytic reasoning (appropriate in second year) using a variety of strategies that would facilitate the development of DR skills. However, they were often using these strategies *intuitively, implicitly* or using explicit strategies but not to their full advantage:

*Sometimes I’m not too sure what things I actually do because it happens on the spur of the moment* (Tutor A)

*I think I teach explicit strategies, but I don’t necessarily teach them explicitly* (Tutor D)

In addition to the five evidence-based strategies from the literature, tutors were found to use a range of other approaches. Several made use of analogy in their general approach, including likening the task to being a detective searching for clues, a car mechanic who understands how everything fits together and works, or a lawyer defending their position in a court of law. Other strategies included data gathering techniques (e.g. ‘drilling’ the presenting symptom), hypothesising broadly (e.g. by applying basic science, tapping into prior knowledge, recognising clusters or patterns provided they know the limitations of using this approach, or using acronyms if necessary), focusing on context and using questions to stimulate thinking.

Tutors reported a variable awareness of the role of the PBL instructional method in facilitating development of DR skills:

*I think the PBL method is very good in that it gives you capacity to teach DR..(but) I do think some people don’t understand the beauty and complexity of using the PBL and so may not be able to utilise it to the fullest* (Tutor H)

Additionally, tutors often felt unsure of the efficacy of the strategies they used:

*I guess I’m uncertain because it’s kind of a woolly area* (Tutor A)

*I suspect they’re not as effective as I’d like them to be* (Tutor C)

*I’d like to say I think they’re very effective but it’s hard to sort of say that with confidence without being able to, in a way, test the student’s diagnostic reasoning capability* (Tutor L)

Several barriers were identified relating to priorities within the curriculum, having inadequate or inappropriate resources, and issues relating to group dynamics.

2. Tutor perceptions of established evidence-based strategies to teach DR

An important theme impacting on the tutors’ approach was the positive influence of the existing evidence base. All five evidence-based strategies discussed at interview were thought to exert positive influences in the preclinical setting in a variety of ways. Firstly, they were thought to improve student knowledge, not only the breadth and depth of knowledge, but also the way knowledge is stored. Secondly, problem representation and ‘comparing and contrasting’ were thought to have direct positive influences on development of DR skills. They helped students to contextualise, to identify relevant information and to learn how to differentiate between similar diseases. Finally, ‘comparing and contrasting’ and metacognition were thought to reduce potential error and bias, including preventing premature closure, and metacognition was thought to help students understand their own limitations, factors that influence thinking and when to seek more information:

*I use comparing and contrasting using defining and discriminating features a lot, because I don’t want them to narrow down, I don’t want them to pigeon-hole a patient too early* (Tutor F)

*It’s important to identify bias in yourself and to recognise there are limitations to your own knowledge* (Tutor H).

However, two of the evidence-based strategies provided some contradictory opinions. Most tutors found the use of prototypes, or classic presentations, useful in their teaching:

*I would use classic presentations to get the basics built correctly* (Tutor L).

However, over one third of the participants had a strong aversion to using prototypes when teaching DR, largely due to concerns about misdiagnosis in atypical presentations, but also because of fears that it would stop students thinking broadly:

*Lots of people do not present like in textbooks .. so if you’re only looking for people with classic presentations you’re going to miss lots* (Tutor F)

*We’ll miss too much if that’s the way we go about doing it* (Tutor B).

Use of metacognition, or thinking about one’s thinking, was encouraged by almost half the participants:

*In a whole range of ways, thinking about how you’re thinking is helpful* (Tutor D)

*It is very useful to reflect back as to why you’re making the decisions you are and what’s led you to that conclusion* (Tutor C).

However, some felt it was difficult or inappropriate in this context:

*I think they’re not at that level* (Tutor F)

*When you try to explain bias to people they think that you’re having a go at them* (Tutor H)

Significantly, tutor beliefs and perceptions also appear to be a barrier. Several participants felt clinical experience was necessary to learn DR and that students can’t apply probabilities and prioritise hypotheses without this experience. In addition, the belief identified in the study that reasoning using prototypes will hinder students thinking broadly and lead to failure to recognise atypical presentations, is a potential barrier to both acquisition of knowledge and development of reasoning skills.

A variety of contextual influences were also identified as barriers. The most frequently cited of these was time constraints”

*Metacognition is the one that, if there’s time pressure, is the first to go. Not to say it’s not important; it’s probably the most important* (Tutor K)

3. Relationships between subject knowledge and DR

A major theme identified was the central role of knowledge. The biggest barrier to teaching and learning DR that was identified by participants was lack of knowledge and this could apply to students or tutors. Participants identified lack of student knowledge of basic and clinical sciences, of medical terminology and of the DR process, as barriers. From a tutor perspective, lack of knowledge about DR, inadequate DR teaching skills, and lack of understanding of the value of certain activities and of what’s appropriate within the curriculum were also identified as barriers.

*DR isn’t a term that is defined that we should be teaching and it’s not a structural entity that is allocated (specific) time at any point in the curriculum: it’s just something you do concurrently* (Tutor J)

Lack of knowledge was also thought to make problem representation (PR) difficult for preclinical students in several ways. Firstly, medical terminology needs to be learned, which is often hard initially. Clarity and agreement in language can also play a role (e.g. the same term can be used slightly differently in different clinical situations) and this was thought to make semantic qualifiers difficult for students without clinical experience:

*The terminology is often not universally agreed on, and varies within different scenarios* (Tutor D)

Inexperienced students also often have difficulty identifying the most important features in a case:

*They have difficulty with [PR] .. I can identify what’s important, but they have to identify what’s important* (Tutor G)

Not surprisingly, education was seen to be a big enabler of the teaching and learning of DR. This included education about the DR process for both students and tutors:

*Maybe I could see those (evidence-based strategies) in a more structured way, and see where they fit into a process to assist our students, and might use them more effectively* (Tutor B).

*Broadening my toolbox would be good* (Tutor E)

*An initial lecture for the students (might help)* (Tutor N).

Faculty professional development about explicit strategies to teach DR and how to use them were thought to be useful, as well as good alignment within the curriculum and guidance for tutors as to what’s appropriate, where, and how to prioritise reasoning with other clinical skills.

4. Student readiness to engage in DR

Certain student learning styles or ways of thinking were also thought to be potential barriers. Several participants thought the concrete thinkers had difficulty while another tutor felt the abstract or creative thinkers struggled. Several participants felt students had different levels of learning maturity and consequently varying capacity to be independent learners and question their thinking. One participant identified being exam focused as a potential barrier:

*Sometimes they’re not so interested in the reasoning process as opposed to what’s going to get them marks in the exam* (Tutor I).

Student confidence was seen to be influential, with both under-confidence and overconfidence identified as barriers (i.e. students staying inappropriately inside or outside their zone of proximal development):

*They’re not confident in their learning and they’re not confident in their own brains, which is really perplexing because they’re so clever* (Tutor H)

*Sometimes a (student) is so sure of themselves that they don’t understand that their certainty about something is also a thinking block* (Tutor H).

Several student characteristics were also thought to be enablers, particularly enthusiasm for what was recognised as being an authentic activity:

*Students relish the opportunity to put it together with DR* (Tutor L)

*Students want to become diagnosticians and they love it* (Tutor M).

Having good concept map- or flowchart-making skills was also seen to be beneficial:

*The map makers will really get it because that particular way of organising your brain is very conducive to deductive thinking* (Tutor A).

## Discussion

The DR process is often poorly understood by clinicians, with the literature suggesting that most clinicians have difficulty articulating steps in the process ( Frick & Bezuidenhout, 2014), which consequently makes the process difficult to teach. The participants in this study were no exception. Our study was conducted within a PBL setting. PBL depends on activation and elaboration of prior knowledge to assist in the development of illness scripts, improving long-term storage and retrieval of knowledge ( Schmidt et al, 2011). However, the study found that tutor awareness is required to maximise the potential opportunities PBL offers, and that, whilst there should be an awareness of atypical presentations, students and tutors should understand that the *focus* in second year should be on *typical* presentations ( Charlin et al, 2007). The findings of this study suggest a need for tutors to be equipped with explicit teaching strategies to help preclinical students develop DR skills. It is suggested that good faculty professional development about DR, explicit DR teaching strategies and how to use them, as well as ensuring staff have good pedagogical skills regarding group dynamics and learning styles, will improve tutor knowledge and address tutor beliefs and perceptions. Improved teaching of explicit DR strategies may then lead to improved student knowledge.

In this study, teaching explicit DR strategies was thought to have a positive influence on the development of DR skills in the preclinical context. There were more perceived advantages than disadvantages to teaching explicit DR strategies. All the explicit strategies discussed (the five evidence-based strategies, as well as other strategies identified by the tutors) should progress students’ reasoning skills if we consider the influences they exert, the barriers to teaching and learning of DR, and the various theories of reasoning. However, some of the evidence-based strategies may need to be used differently in the preclinical context. An example of this is PR, which may need to be broken down into earlier steps of learning medical terminology and how to recognise the important information in the clinical scenario. PR should be tailored to the individual student, with some working on these earlier steps, whilst others might be attempting the full 1-2 sentence summaries. Further research is required to assess modification of the existing evidence-based strategies for the preclinical setting. Metacognitive techniques were also found in this study to be very useful. Metacognitive questioning, provided it is presented as an essential skill that good clinicians use daily and taught within a supportive environment, will improve insight and skill in reasoning ( Kassirer, 2010). Challenging students to defend their opinions will facilitate better illness script formation by strengthening linkage of causal mechanisms and clinical features ( Chamberland et al, 2015). It can also be used to demonstrate the student’s reasoning process and identify learning deficits ( Dhaliwal, 2013). Teaching an awareness of bias and specific strategies to eliminate bias (e.g. comparing and contrasting hypotheses, reflection on one’s thinking) can improve data collection and minimise error ( Nendaz & Perrier, 2012).

Knowledge plays a crucial role in clinical reasoning ( Norman, 2005), with good clinical reasoning dependent on access to knowledge and appropriate application of knowledge. Developmental stages in CR relate to a mix of formal and experiential knowledge ( Schmidt et al, 1990). Preclinical medical students are acquiring vast amounts of formal or theoretical knowledge and helping them store that knowledge in a readily retrievable form, using strategies informed by script theory, should ultimately improve reasoning skills. According to Charlin (2007), illness scripts start developing early and are continually refined. Students should initially be taught using typical cases and then less typical cases can be introduced to develop the script. Students who start developing basic illness scripts early, can activate and enrich them when they encounter real patients in clinical placements (Custers et al, 2015). Using prototypes in preclinical years should theoretically increase student knowledge of specific diseases, assist with early script formation and help in recognising similarities between diseases and the importance of identifying discriminating features. Encouraging students to look for and learn clusters of symptoms will not only increase knowledge but may help students either develop or activate illness scripts. All strategies that encourage students to link basic science and clinical features (e.g. using concept maps or flow charts) and consider enabling factors (e.g. looking at context, probabilities, demographics, aetiological factors) should strengthen students’ understanding and facilitate development of illness scripts. Further research is also required in these areas.

Teaching time-poor students that the DR process is important in getting the right diagnosis, and tapping into their enthusiasm for an authentic activity, should help them prioritise the development of reasoning skills. Guidance from faculty regarding priorities should also address time constraints and the place of DR within the curriculum.

One limitation of this study is that it looked at the experience of a group of tutors from only one university and one year of a graduate-entry, hybrid problem-based learning degree, and all the tutors were clinical doctors. This may limit the generalisability to other institutions. Similar issues to those explored in this study could be explored in other preclinical contexts e.g. with non-medical PBL facilitators and first year students. Medical schools with non-medical PBL facilitators could be expected to have additional specific professional development needs to upskill their tutors in the clinical reasoning processes of medical doctors. There may also be potential implications for professional development in the clinical setting, for example how are clinical tutors to build on the diagnostic reasoning skills acquired in the preclinical years? What training will the tutors require to further this basis? Another weakness of the study was the lack of direct observation and tutors being often unable or unwilling to provide definitive statements about the effectiveness of the DR strategies they were teaching. Observing tutors and students navigating DR in PBL tutorials and investigating the efficacy of explicit DR strategies in the preclinical setting could be areas for future research.

## Conclusion

This study adds to our understanding of the teaching and learning of DR in the preclinical context, a relatively under-researched area in the literature. Tutors of preclinical medical students perceive a definite role for teaching explicit DR strategies in this context. However, they often teach DR intuitively, implicitly or using explicit strategies but not to their full advantage. Some evidence-based strategies may need to be tailored specifically for use in the preclinical context and a range of strategies need to be taught to minimise the risks of failing to think broadly enough and prevent bias. Strategies used should aim to promote acquisition, organisation and storage of knowledge (based on understanding of script theory) and to develop metacognitive awareness. Barriers to teaching and learning of DR skills include lack of knowledge, tutor beliefs and perceptions, student thinking styles and contextual factors. These barriers can be overcome by well-designed tutor professional development aimed at increasing tutor knowledge on the theoretical basis of DR and upskilling in a range of evidence-based pedagogies for teaching DR.

## Take Home Messages

• The use of explicit strategies by tutors enhances the development of diagnostic reasoning skills in preclinical medical students
• A range of strategies should be used by tutors, including those aiming to improve organisation and storage of knowledge, as well as metacognitive strategies
• Some strategies used by tutors in the clinical setting may need to be modified for preclinical students
• Use of explicit strategies by tutors can help overcome some of the barriers to teaching and learning of DR skills, especially lack of knowledge
• The provision of professional development of tutors, which specifically addresses the complexity required in addressing these learning needs, is essential.

## Notes On Contributors

Dr Jane Keir is a PBL tutor and GP Clinical Supervisor at the University of Notre Dame Australia (UNDA), School of Medicine Sydney. She also facilitates a postgraduate unit in Teaching Clinical Reasoning for the UNDA School of Medicine, Fremantle. Her research interest is in clinical reasoning.

Dr Shannon Saad is the Head of Communications and Clinical Skills domain at the University of Notre Dame (UNDA), Sydney. This role oversees the development and delivery of those aspects of the curriculum that relate to clinical practice. Research interests include healthcare communication and clinical reasoning.

Dr Lorna Davin is a Senior Lecturer in Medical Education at the University of Notre Dame Australia (UNDA), Fremantle. She is Program Coordinator for the UNDA Health Professional Education postgraduate program. Her research interests are in compassion in healthcare, for patients and their doctors.
