# Supplementary material for: Exploring tutor perceptions and current practices in facilitating diagnostic reasoning in preclinical medical students: Implications for tutor professional development needs
Source: MedEdPublish (2016). 2018 May 22;7:106. [Version 1] doi: 10.15694/mep.2018.0000106.1 (PMC10699380; doi:10.15694/mep.2018.0000106.1)
Supplement: Peer review history

# Reviewer report: Reviewer response for version 1

**DOI:** 10.21956/mep.19573.r29041

**Published:** 2018-05-29

**Authors:** Subha Ramani

**Affiliations:** 1 Harvard Medical School, Brigham and Women's Hospital

This review has been migrated. The reviewer awarded 5 stars out of 5 I thoroughly enjoyed reading this article. Diagnostic reasoning is very close to my own professional role as a clinician educator and all of us find it challenging to diagnose the DR skills of learners and to demonstrate and role-model our skills. The theoretical underpinnings of DR were very well described and I learned a lot reviewing this background. Using these frameworks, a qualitative approach to making this inquiry is very appropriate. Rich insights have been obtained though the number of participants is small. This inquiry can be extended further to add to the field and inform educators and educationalists about this topic.It is easy to ask teachers to think out loud, but they need guidance in framing their think out loud techniques to effectively model and teach and assess CR and DR.

Reviewer Expertise:

NA

No decision status is available

# Reviewer report: Reviewer response for version 1

**DOI:** 10.21956/mep.19573.r29043

**Published:** 2018-05-22

**Authors:** Megan Anakin

**Affiliations:** 1 University of Otago

This review has been migrated. The reviewer awarded 4 stars out of 5 I too found this clearly written article informative and thought-provoking. A good summary of clinical/diagnostic reasoning provides the background to the focus of the study. The research question is addressed appropriately by the chosen methods. By examining the perspective of tutors, this study adds to our knowledge about how clinical/diagnostic reasoning is taught. The authors’ findings are also consonant with other studies about this topic. It is important that this study is published and read because we have yet to amass sufficient evidence to conduct a thorough review of this topic. I was left pondering how challenging it is to develop a well-defined medical curriculum, even in a pre-clinical setting, especially when the tutors are clinicians who have expertise as practitioners and who may not have yet developed expertise as educators.

Reviewer Expertise:

NA

No decision status is available

# Reviewer report: Reviewer response for version 1

**DOI:** 10.21956/mep.19573.r29042

**Published:** 2018-05-22

**Authors:** Klaus von Pressentin

**Affiliations:** 1 Stellenbosch University

This review has been migrated. The reviewer awarded 4 stars out of 5 This study explores the teaching of diagnostic reasoning at the level of preclinical medical students from the perspectives of 14 tutors engaged in a PBL curriculum. The authors made a clear link between the theoretical considerations and the challenges of making the teaching of diagnostic reasoning explicit. During the interviews, it became clear that the tutors employed intuitive techniques and half of the interviewees struggled to articulate how they approached the process of teaching diagnostic reasoning, a key component of clinical reasoning aimed at reaching a diagnosis. The themes which emerged supported the existing literature, which highlight the poor understanding of experienced clinicians of how to break the process into teachable steps. This study does make the recommendation for tutors in pre-clinical PBL-oriented programmes to focus on typical presentations, as both students and tutors lack core knowledge components: students have to visualize the important aspects of clinical scenarios without any real life exposure, and tutors seem to lack knowledge of explicit techniques of teaching diagnostic reasoning. Diagnostic reasoning activities present important opportunities for building enthusiasm and for linking theoretical knowledge to application in typical clinical scenarios or prototypes. The authors highlighted some relevant limitations, including the potential lack of generalizability to other contexts, non-medical facilitators and students, and non-PBL programmes. This study was explorative in nature, yet appropriately grounded in the relevant underlying learning and clinical reasoning theories. The conclusions emphasise the need for more research in the application of diagnostic reasoning skills in the pre-clinical curriculum. Faculty development activities should be aimed at equipping tutors with the necessary theoretical knowledge and evidence-based tools for making this facilitation of learning more explicit. The study inspired this reviewer to reflect on his own practice and to gain a deeper understanding of the cognitive processes behind clinical reasoning and diagnostic reasoning. These reasoning skills and reflective approach towards understanding the underlying cognitive processes should be nurtured in an explicit manner from the pre-clinical years onward, as these tools will also apply to experienced practitioners who may need to reflect on how best to deconstruct their own reasoning when engaging with students.

Reviewer Expertise:

NA

No decision status is available
